# A rare presentation of metastatic papillary thyroid carcinoma mimicking a branchial cyst in a 35-year-old female: A case report

**DOI:** 10.1016/j.ijscr.2024.110661

**Published:** 2024-11-26

**Authors:** Abdullah Fadhel Almusallam, Mosab Tareq Atmeh, Raad Y. Al Tahat, Chaima Karchoud, Ahmed Smadi, Mousa Tarek Atmeh

**Affiliations:** a Kuwait Ministry of health, Kuwait; b Jordan university of science and technology, Jordan; c Department of Radiology, Royal Medical services, Jordan; d Jordan Hospital, Jordan; e Department of otolaryngology, Royal Medical services, Jordan; f Department of oncology, Royal medical services, Jordan

**Keywords:** Papillary thyroid carcinoma, Branchial cyst, Metastasis, Neck mass, Thyroidectomy

## Abstract

**Introduction and importance:**

Neck masses are common in clinical practice, with branchial cysts presenting as painless, slow-growing lateral neck masses. Metastatic involvement of branchial cysts by thyroid carcinoma is rare. Papillary thyroid carcinoma (PTC) represents 75 %–85 % of thyroid cancers and often presents with a neck mass. This case report describes a rare presentation of metastatic PTC mimicking a branchial cyst in a 35-year-old female, emphasizing the importance of thorough evaluation.

**Case presentation:**

A 35-year-old female presented with a three-year history of a slowly enlarging right submandibular neck mass. She was otherwise healthy, with no significant family or medical history. Physical examination revealed a well-defined, non-tender mass in the right anterior neck, measuring 3.4 cm. No lymphadenopathy or thyroid nodules were detected. MRI revealed an enlarged cystic lymph node. Fine-needle aspiration initially suggested a branchial cyst, but histopathology after excision confirmed metastatic PTC. Subsequent thyroid ultrasound showed a 3 mm hypoechoic nodule in the right lobe, classified as TI-RADS IV. Total thyroidectomy was planned.

**Clinical discussion:**

Branchial cysts are typically benign, but this case highlights the possibility of malignancy. Excision and postoperative biopsy are essential for diagnosis, as seen in this case, leading to a total thyroidectomy plan.

**Conclusion:**

Neck masses can conceal malignancies, including metastatic PTC. This case underscores the importance of thorough pathological evaluation to ensure appropriate management.

## Highlights

• Unusual Presentation: A rare case of metastatic papillary thyroid carcinoma (PTC) mimicking a branchial cyst in a 35-year-old female.
• Diagnostic Challenges: FNA suggested a benign branchial cyst, but histopathology after excision revealed metastatic papillary thyroid carcinoma (PTC).
• Importance of Imaging: Preoperative MRI showed an enlarged cystic lymph node, and thyroid ultrasound detected a TI-RADS IV suspicious nodule.
• Treatment Approach: Excision of the cyst followed by total thyroidectomy to remove the primary malignancy and prevent further spread of disease.
• Clinical Significance: Underscoring the need for thorough evaluation of neck masses, even when benign conditions like branchial cysts are suspected.

## 1 Background

Branchial cleft cysts are congenital anomalies which develop in utero, most commonly arising from the second branchial cleft. The cystic cavity can form a potential space which can harbor infection and, in rare cases, malignant spread of primary tumours [1].

PTC is characterized by its indolent growth and often asymptomatic nature, which can lead to delayed diagnosis. Studies suggest that up to 60 % of thyroid cancers may be non-palpable at the time of diagnosis, frequently identified incidentally during imaging for unrelated issues. This can contribute to the misclassification of neck masses as benign, particularly in patients who do not present with typical symptoms such as pain, dysphagia, or hoarseness. In our case, the absence of such symptoms may have led to the initial misdiagnosis, highlighting the importance of thorough evaluation, including imaging and histopathological analysis, in any persistent neck mass.

PTCs are common thyroid tumours with metastatic potential. These tumours share an occult clinical picture consistent with asymptomatic congenital neck masses [2]. Many instances are non-palpable “incidentalomas” evading routine thyroid examinations only to be detected serendipitously through unintentional imaging. In fact, autopsy studies have shown 30 %–60 % of patients have non-palpable thyroid masses; the majority (87 %) are without malignant potential and can be safely monitored with physical exam and routine imaging alone [3]. Rapidly enlarging head and neck masses, masses with a diameter greater than 1 cm, vocal cord paralysis, hoarseness, cervical lymphadenopathy, excessive childhood exposure to radiation, and family histories pertinent for familial cancers are suspect for malignancy [4]. Notwithstanding, overall survival is as high as 90 % at 10 years with prompt treatment initiation [5]. Age at diagnosis and delays in identifying metastatic spread are both associated with poorer overall outcomes [6]. PTC metastasis within the confines of a branchial cleft cyst has been reported in the literature, albeit infrequently [7].

## 2 Introduction

Neck masses are a prevalent clinical challenge, often stemming from various benign or malignant conditions. Among these, branchial cysts—congenital anomalies originating from incomplete closure of the branchial apparatus—are commonly encountered, typically presenting as asymptomatic, slow-growing lateral neck masses. Although generally benign, the possibility of malignant transformation within these cysts is a critical aspect that warrants attention.

Congenital cervical anomalies are important to consider in the differential of head and neck masses in children and adults. These lesions can present as palpable cystic masses, infected masses, draining sinuses, or fistulae [8].

Papillary thyroid carcinoma (PTC), the most common type of thyroid cancer, frequently presents as a neck mass due to lymphatic spread. The insidious nature of PTC often results in it being misdiagnosed, as many patients remain asymptomatic until late in the disease process. Interestingly, PTC can metastasize to lymph nodes or other structures, and its presentation can occasionally mimic benign entities, such as branchial cysts. This rare overlap poses significant diagnostic challenges, emphasizing the need for a high index of suspicion in cases where clinical findings deviate from the expected patterns.

This case report describes a 35-year-old female with a metastatic PTC that initially presented as a branchial cyst, highlighting the importance of comprehensive evaluation and the potential for malignant disease masquerading as benign pathology in the neck.

## 3 Case report

A 35-year-old female patient, married with four children, presented to the ENT clinic with a more than three-year history of a slowly growing right submandibular neck mass. She had no significant past medical or surgical history and reported no symptoms such as dysphagia, dyspnea, or voice changes. The patient was otherwise healthy, and her family history was unremarkable for thyroid or other cancers.

## 4 Clinical findings

Physical examination revealed a well-defined, non-tender mass located in the right anterior triangle of the neck, measuring approximately 3.4 cm in diameter. There were no palpable cervical lymphadenopathies or thyroid nodules on examination. No signs of local invasion or skin changes were observed.

## 5 Timeline

• **3+ years before presentation**: Onset of a slowly growing right submandibular neck mass.
• **Presentation to ENT Clinic**: The FNA showed a well-defined lesion, initially suspected to be a branchial cyst.
• **In January 2024**: Cyst aspirated; surgical excision planned.
• **February 2024**: Surgical excision of the Right branchial cyst.
• **Post-surgery in February 2024**: Histopathology revealed metastatic papillary thyroid carcinoma.
• **In February 2024**: Thyroid ultrasound showed a 3 mm hypoechoic nodule in the right thyroid lobe (TI-RADS IV).
• **In February 2024**: TSH, T3 and T4 were normal.
• **In March 2024**: The patient was planned for total thyroidectomy.

## 6 Therapeutic intervention

The patient underwent surgical excision of the suspected branchial cyst, which revealed metastatic papillary thyroid carcinoma. Based on these findings, a total thyroidectomy was planned to remove the primary thyroid lesion and prevent further metastatic spread. The patient is also planned for radioactive iodine ablation to target any residual thyroid tissue and potential microscopic disease as well as TSH suppression therapy with thyroxine in order to reduce the risk of recurrence.

## 7 Diagnostic assessment

**Figs. 1 and 2:**
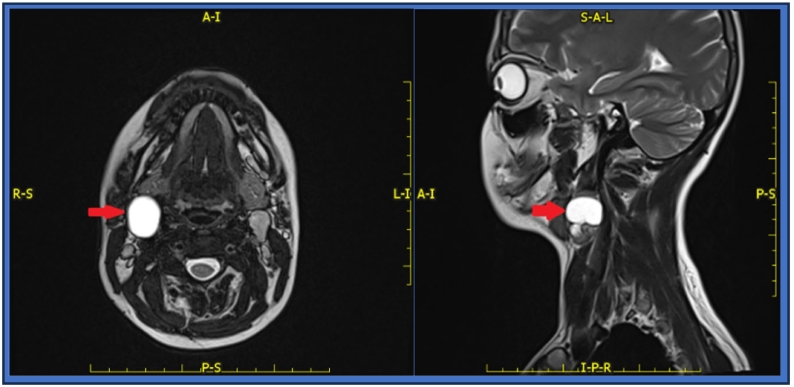

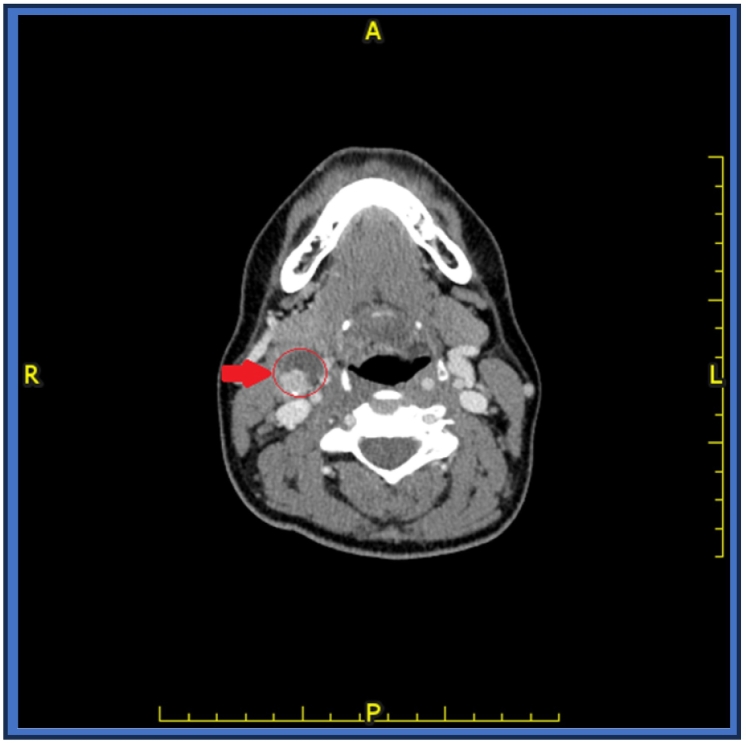
respectively: An axial and a sagittal T2 weighted image (T2WI) show an enlarged cystic lymph node on the right side of the neck at group IIA.

Preoperative imagining including Magnetic Resonance Imaging (MRI) of the head and neck shows an enlarged cystic lymph node on the right side of the neck at group IIA as seen in Figs. 1 and 2 below.

**Fig. 3:**
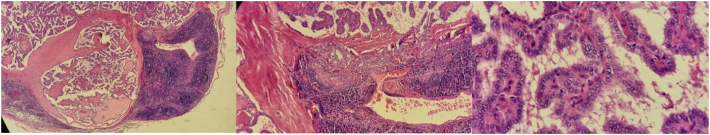
Axial post contrast neck CT scan shows group IIA enlarged cystic lymph node with enhanced soft tissue component and calcific focus.

A post contrast neck CT scan shows an enlarged cystic lymph node with enhanced soft tissue component and calcific focus at group IIA as seen in Fig. 3 below.

**Fig. 4:**
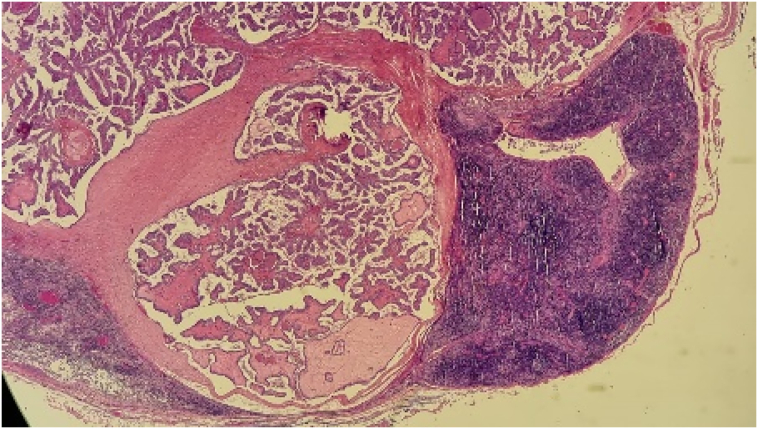
2.5× Magnification.

**Fig. 5:**
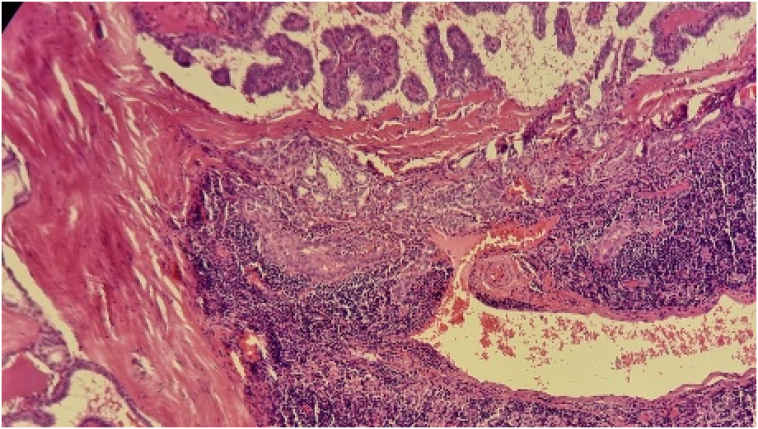
10× Magnification.

**Fig. 6:**
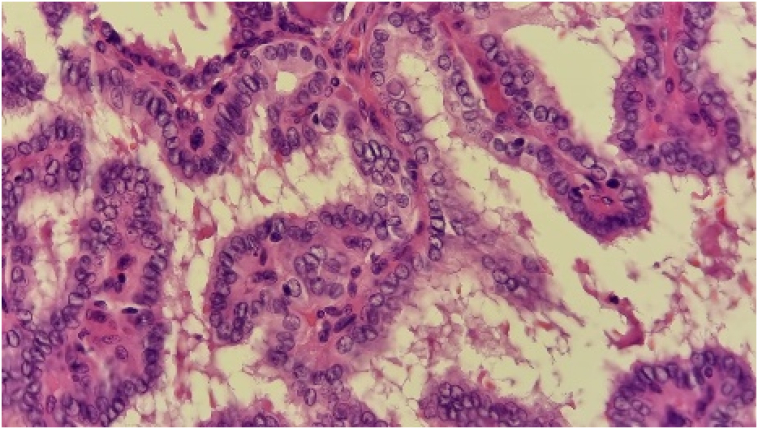
40× Magnification.

Fine-needle aspiration (FNA) initially suggested a branchial cyst. However, following surgical excision of the cyst, histopathology revealed metastatic papillary thyroid carcinoma. Gross examination of the cyst showed a 3.4 × 2 × 2 cm lesion containing brownish fluid with areas of papillary projections. Microscopic examination (Seen in the Fig. 4, Fig. 5, Fig. 6 below) confirmed metastatic carcinoma.

Subsequent thyroid ultrasound revealed a 3 mm hypoechoic solid nodule in the right thyroid lobe, classified as a TI-RADS IV lesion, suggestive of a suspicious malignancy. No other thyroid lesions or signs of capsular breach were observed No suspicious lymphadenopathy was identified on imaging, except for a few reactive lymph nodes in the right cervical area near the surgical site. Thyroid function tests were within normal limits. The patient is scheduled for total thyroidectomy. Postoperative management will include monitoring for complications, such as hypocalcaemia or recurrent laryngeal nerve injury, and follow-up imaging to assess for any residual disease or recurrence. Long-term management will involve thyroid hormone replacement therapy and potentially radioactive iodine therapy, depending on the final histopathological findings.

It is worth it to highlight that the initial MRI and FNA findings suggested a benign branchial cyst, however, the final diagnosis of metastatic PTC was made after surgical excision and histopathological examination. This emphasizes the importance of excisional biopsy in cases with ambiguous imaging findings, as it is crucial for definitive diagnosis and guiding treatment, and to always keep in mind the possibility of a malignant mass even in the presence of benign features.

## 8 Discussion

The occurrence of papillary thyroid carcinoma (PTC) metastasizing within a branchial cyst is an exceedingly rare phenomenon that underscores the complexities of diagnosing neck masses [7]. While branchial cysts are often considered benign, their potential for harboring malignant processes, particularly in young adults, necessitates a vigilant approach. In this case, the patient's long-standing neck mass—initially perceived as a cosmetic concern—was ultimately revealed to be a manifestation of metastatic disease.

PTC metastasis within the confines of a branchial cleft cyst has been reported in the literature, albeit infrequently [7]. A review of these cases revealed a similar set of circumstances. That is, the patient underwent excision of a branchial cleft cyst with unexpected histological staining patterns consistent with PTC. Radiologic imaging was generally benign with purported post-hoc evidence suspicious of nonconformity in only one case. Atypical characteristics for second branchial cysts, such as wall thickening, enhancement, dystrophic calcifications, and development of septations, may hint at complex branchial cleft cyst pathology [7]. Even with these findings, the overlap between other neoplastic, infectious, and inflammatory etiologies may still require excision with post-excisional tissue analysis. In each of the aforementioned cases, tissue analysis led to the subsequent identification of primary thyroid lesions in the absence of discernible signs and symptoms.

Branchial cleft cysts are congenital anomalies which develop in utero, most commonly arising from the second branchial cleft. They are often asymptomatic lateral neck masses but can enlarge and become symptomatic in the setting of infection. The cystic cavity can form a potential space which can harbor infection and, in rare cases, malignant spread of primary tumours [1].

PTCs are common thyroid tumours with metastatic potential. These tumours share an occult clinical picture consistent with asymptomatic congenital neck masses [2]. Many instances are non-palpable “incidentalomas” evading routine thyroid examinations only to be detected serendipitously through unintentional imaging. In fact, autopsy studies have shown 30 %–60 % of patients have non-palpable thyroid masses; the majority (87 %) are without malignant potential and can be safely monitored with physical exam and routine imaging alone [3]. Rapidly enlarging head and neck masses, masses with a diameter greater than 1 cm, vocal cord paralysis, hoarseness, cervical lymphadenopathy, excessive childhood exposure to radiation, and family histories pertinent for familial cancers are suspect for malignancy [4]. Notwithstanding, overall survival is as high as 90 % at 10 years with prompt treatment initiation [5]. Age at diagnosis and delays in identifying metastatic spread are both associated with poorer overall outcomes [6].

Preoperative imaging, including ultrasound and MRI, can aid in the assessment of thyroid nodules and potential metastatic disease. However, imaging alone may not always distinguish benign from malignant lesions. FNA and histopathological analysis remain the gold standards for diagnosis.

Surgical excision is the primary treatment for metastatic PTC, with total thyroidectomy indicated to address the primary thyroid malignancy and prevent further metastatic spread. Postoperative management should focus on surveillance for recurrence and thyroid hormone replacement therapy.

Fortunately, in this case, the patient sought medical care due to cosmetic concerns related to the branchial cleft cyst. Had the patient not experienced these concerns, they might not have pursued treatment, as asymptomatic patients are less likely to seek medical attention. Consequently, the diagnosis and management of the underlying malignancy in this patient could have been delayed without the cosmetic motivation. Patients with similar socioeconomic backgrounds and health conditions may face increased risk since branchial cleft cyst excision is often considered elective unless symptoms are present, which can lead to delays in diagnosis and worsen outcomes. The limited data on branchial cleft cysts, especially those with concurrent metastatic involvement, presents a challenge for further research. As more cases are documented, future meta-analyses may help to shed light on this rare association.

## 9 Conclusion

Neck masses are commonly encountered in clinical practice and can arise from a variety of benign or malignant conditions. While branchial cysts are typically benign congenital lesions, clinicians should remain vigilant for rare cases where malignant processes, such as metastatic papillary thyroid carcinoma, may be involved. This case underscores the importance of thorough diagnostic evaluation, including histopathological examination, to ensure accurate diagnosis and appropriate treatment. Continued research and documentation of similar cases may provide valuable insights into the rare interplay between benign and malignant neck masses, ultimately enhancing clinical practice and patient care. Due to the rarity of this condition and the potential for recurrence, long-term follow-up is advised.

In summary, this case emphasizes the importance of maintaining a high degree of suspicion for malignancy in neck masses, even when they present with typical benign characteristics. The diagnostic challenges presented by such cases serve as a reminder of the need for comprehensive evaluation and the potential consequences of delayed diagnosis. This case affirms the importance of thorough diagnostic evaluation for neck masses, even when initial assessments suggest a benign mass, physicians should remain vigilant and consider malignancy in the differential diagnosis and ensure follow up of these cases, staying alert to any red flags that might arise, as this approach can significantly impact patient's management and outcomes.

## Methods

This case report has been written in accordance with the SCARE 2023 guidelines [9].

## CRediT authorship contribution statement

Abdullah Fadhel Almusallam: literature review, writing, editing, manuscript drafting.

Mosab Tareq Atmeh: Literature review, writing, editing, and manuscript drafting.

Raad Y.Altahat: Patient management planning, assisted in imaging acquisition and interpretation, contributing to the diagnostic process and management of the case.

Chaima Karchoud: Literature review, writing, and editing.

Ahmed Smadi: patient management planning, critical review, supervision, final approval.

Mousa T.Atmeh: patient management planning, critical review.

## Informed consent

Written informed consent was obtained from the patient for the publication of this case report and any accompanying images. The patient was informed that no identifying information would be disclosed.

## Ethical approval

According to the policies of the institute where the case report was conducted, ethical approval is not required for case reports, and therefore, they are exempt from ethical approval.

## Guarantor

Abdullah Fadhel Almusallam.

## Patient perspective

The patient expressed concern about the possibility of cancer and was initially reassured by the diagnosis of a benign branchial cyst. However, she was anxious about the unexpected finding of metastatic papillary thyroid carcinoma and the need for further surgery. She has been informed about the prognosis and the importance of follow-up care, and she remains committed to her treatment plan.

## Source of funding

No funding was received for the preparation of this case report.

## Declaration of competing interest

The authors declare no conflict of interest regarding the publication of this case report.
